# Study on the Classification, Causality, Preventability and Severity of Adverse Drug Reaction Using Spontaneous Reporting System in Hospitalized Patients

**DOI:** 10.3390/pharmacy6040108

**Published:** 2018-09-29

**Authors:** Siraj Sundaran, Anjali Udayan, Keerthi Hareendranath, Basil Eliyas, Babu Ganesan, Ashik Hassan, Rajesh Subash, Vishnu Palakkal, Mohammed Saji Salahudeen

**Affiliations:** 1 Devaki Amma Memorial College of Pharmacy, Kerala University of Health Sciences, Malappuram, Kerala 673634, India; anjaliudayan1@gmail.com (A.U.); Keerthi.h@outlook.com (K.H.); basileliyas4@gmail.com (B.E.); gbabu73@gmail.com (B.G.); vishnu.p@outlook.com (V.P.); 2 PVS Hospital Pvt Ltd., Calicut, Kerala 673002, India; ashik.h@outlook.com (A.H.); rajesh.s@outlook.com (R.S.); 3 Division of Pharmacy, School of Medicine, University of Tasmania, Hobart 7001, Australia; mohammed.salahudeen@otago.ac.nz

**Keywords:** adverse drug reactions, spontaneous reporting, causality, ADR, severity

## Abstract

Hospital-based adverse drug reaction (ADR) monitoring and reporting programs intend to identify and quantify the risks associated with the use of medicines. To examine the causality, preventability and severity of ADR in a hospital setting; a prospective cohort study on spontaneous ADR reporting was conducted from December 2015 to May 2016. Incidence of ADRs, causality, type, severity and preventability were assessed using necessary assessment scales. The study included 3157 hospitalized individuals, in whom 51 ADRs were detected among 49 patients. The overall incidence of suspected ADRs was found to be 1.6%. According to the causality assessment, most of the ADRs reported were probable (*n* = 26, 51.0%), and type A (augmented/pharmacological) reactions (*n* = 39, 76%) were the most common type of ADR found. The majority of ADRs were moderate to severe (*n* = 35, 68.6%), of which 37.3% were found to be potentially preventable. Predictability was observed in 28 (54.9%) reported ADRs. The prescribed medicines most frequently associated with ADRs were antibiotics, antiepileptics and antihypertensives. This feasibility study was able to highlight the clinical pharmacist’s role in ADR monitoring service and create awareness about the way it could be done to promote safer medication use. Similar ADR reporting programs are necessary to educate and to improve awareness among healthcare professionals in some countries.

## 1. Introduction

Medications are being widely prescribed for various medical conditions, and the selection of a medicine is often based on the benefit-risk ratio. Adverse drug reactions (ADRs) are considered one of the leading causes of morbidity and mortality [1,2]. It is postulated that 5–8% of all hospitalized individuals experience serious ADRs and 10% of the hospital costs are related to ADRs [3,4,5]. Hospitalization and complications during hospitalization, such as prolonged hospital stay and increased healthcare costs, are the burdens mainly associated with ADRs [6,7]. According to the World Health Organization (WHO) [8], “ADR is a noxious and unintended response that occurs at doses normally used in humans for prophylaxis, diagnosis or therapy of disease, or for the modification of physiological function”. This definition eliminates therapeutic failure, overdose, medication abuse, noncompliance, and medication error [9,10]. Numerous factors influence ADR susceptibility, including polypharmacy, age, disease severity and the type of medicines prescribed [11].

ADRs are considered an unnecessary cause to an already burdened healthcare system and are usually preventable. Although many of the regulatory agencies obligate ADR monitoring, it is not extensively accomplished in Indian hospitals. In some countries, ADRs are under-reported and undisclosed due to lack of drug monitoring and prioritization of medication safety [12]. The extent of medication use differs across countries, and the findings from each population cannot be generalized [13,14]. Since the number of medications and their usage have increased recently, the early detection of ADRs is essential to monitor both known and unknown effects of a medication [15]. A recent systematic review reported that the median incidence rate of ADRs among Indian hospitals is high (12.9%) [11].

Hospital-based ADR reporting programs can provide valuable information about the potential issues associated with medication usage in that institution. In addition, the ability to detect rare ADRs and to generate new signals would enhance a sound pharmacovigilance system in the country by revealing unusual or rare ADRs to the Indian population. However, physicians are still unaware of ADR reporting and monitoring services, as many untoward adverse incidents are unrecognized in an Indian hospital setting [16]. Conducting prospective studies allows more precise reporting of medication-related history and symptoms to assess the causality of ADRs. Hence, the aim of this study was to examine the ADRs in a tertiary care hospital and assess the type, causality, preventability and severity of the ADRs. The present study was intended to monitor ADRs in a tertiary care hospital where the clinical pharmacy services has been already established.

## 2. Methods

This prospective cohort study was conducted in the inpatient medical wards at PVS Hospital (P) LTD, Calicut, Kerala, India, a 350-bed tertiary care teaching hospital. The study duration was six months, from December 2015 to May 2016. A prospective spontaneous ADR reporting method was followed for the study. Since 1960, WHO is using spontaneous reporting systems, also called ‘early warning’ systems [17] which is widely recognized in many countries.

Awareness about the ADR monitoring and reporting was given to the physicians, surgeons, nurses, pharmacists and allied medical staff of the hospital. Routine case-sheet review and medication order review were done by the clinical pharmacists. For each patient, the prescribed medications were noted and possible occurring ADRs were listed from relevant references. Each day, the patients were interviewed and checked for the identified ADRs and reported the classification, causality, preventability and severity using appropriate measures. The ADR report of the concerned patient was submitted to the physician or surgeon in-charge in the form of ‘thank you note’ (Supplementary Table S1). The ADR reports were finally documented in the ADR documentation forms (Supplementary Table S2). ADR notification forms were given to all the inpatient units, and these helped with reporting of ADRs at times when the clinical pharmacists were not available during the occurrence of an ADR (Supplementary Table S3).

The reported ADRs on the notification forms, after been confirmed by the physician-in-charge, were assessed for causality using Naranjo’s algorithm scale [18], type of ADR using Wills and Brown classification [9], preventability using Modified Schumock and Thornton scale [19] and severity using Modified Hartwig and Siegel scale [20]. Predictability was assessed based on the incidence rate of the reported ADRs as per product information and relevant literatures. The completed ADR documentation was countersigned by the physician-in-charge. We led individual causality assessments to improve the accuracy of ADRs.

### 2.1. Statistical Analysis

All descriptive statistical analysis will be performed using SPSS version 24 (IBM Corp., Armonk, NY, USA). Rate of ADR occurrence and total number of inpatients during the study period was compared to identify the overall incidence of ADRs. The Chi-square test was used to analyze categorical data.

### 2.2. Ethical Approval

The institutional ethics committee of PVS Hospital (P) Ltd., Calicut, Kerala, India approved clearance for this study.

## 3. Results

The study recorded 3157 inpatient admissions during the study period, of which 51 ADRs were detected among 49 patients. The overall ADR incidence rate was 1.6%. Females experienced a higher incidence of ADRs (1.6%) than males (1.5%). The male to female ratio according to occurrence of ADRs was 0.8. Adult (19–64 years) patients experienced a higher number of ADRs (*n* = 23, 46.9%) than older people (*n* = 21, 42.9%) aged 65 and above. The least number of ADRs were found in young patients (*n* = 5, 10.2%) aged 18 years or younger. A summary of patient characteristics is given in Table 1.

Particulars on the classification and other assessments of ADRs are given in Table 2. In relation to the Wills and Brown classification, 39 (76.5%) ADRs were type A (augmented) reactions and 10 (19.6%) were type H (hypersensitivity) reactions. According to the Naranjo’s algorithm scale, 26 (51.0%) reactions had probable relation to the suspected medications and 25 (49.0%) had possible relation to the suspected medications. The overall severity assessments showed that the majority of the reactions reported were moderate (35, 68.6%), followed by mild (11, 21.6%) and severe (5, 9.8%) reactions. Assessment on preventability showed that 19 (37.3%) ADRs were probably preventable, 17 (33.3%) were not preventable and 15 (29.4%) were definitely preventable. The study observation showed that 28 (54.9%) of ADRs were predictable and rest of the 23 (45.1%) were unpredictable.

Among 15 (29.4%) patients, the suspected medication was stopped and substituted with another medication for the same indication. In addition, another medication was added to relieve the ADR in 9 (17.7%) patients, and the dose of suspected medication was reduced to ameliorate the symptoms in 12 (23.5%) patients. Among the observed ADRs, 29.4% of the suspected drugs were discontinued, followed by another 29.4% of medications being withdrawn, and an additional treatment/antidote given to manage ADRs. The outcome of ADR management showed 58.8% were recovered and 41.2% were in the process of recovering phase. The most common medications causing ADRs and their reactions are given in Table 3. Antibiotics were associated with most number of the ADRs reported (*n* = 14, 27.5%), with ciprofloxacin (*n* = 2, 3.9%) and metronidazole (*n* = 2, 3.9%) associated with the highest number of ADRs.

## 4. Discussion

This study examined and explored the types of ADRs and their causality, preventability and severity among hospitalized individuals. The evidence from the current study found an overall incidence rate of ADR to be 1.6%, where females showed a higher incidence rate of ADR than males. However, a meta-analysis conducted by Lazarou et al. [4] found a 15.1% incidence rate of ADR among the hospitalized patients. One of the main reasons for a lower incidence rate in our study is that we followed spontaneous reporting system while Lazarou et al. [4] identified ADRs using the prospective surveillance method. Some major flaws of spontaneous reporting system are incompleteness of data collection in terms of quality and quantity, underreporting, lack of established risk factors, reporting bias, and death due to an ADR may be recorded incomplete [21]. A review of observational studies in Europe reported a lower incidence rate of ADR (3.6%) amid all hospitalizations (median; mean 4.6%) in 22 studies among unselected patient populations [22]. A study conducted from Nepal also reported a lower prevalence of ADR (0.86%) [23].

One of the limitations of this study was the use of spontaneous reporting system that leads to lower rates of ADR reporting. According to the literature, under-reporting is one of the major limitations of spontaneous reporting system followed by variable quality of the reported data and sparse information on medication exposure [21,24,25,26]. Use of Naranjo scale was identified as another limitation of this study. Recent studies have questioned the use of Naranjo’s algorithm and compared new instruments against the superseded Naranjo scale. Adapted from the Naranjo algorithm, recently a new assessment method was developed known as the Liverpool ADR Causality Assessment Tool (LCAT) showed high interrater agreement when used by its developers [27,28].

Our study’s findings show that older patients experience higher incidence of ADRs (2.2%) when compared to the adult patients (1.5%) and younger patients (0.7%). Likewise, numerous studies have found an up to two-fold increase in the number of older people being hospitalized because of ADR-related problems compared to their younger counterparts [29,30,31].

Results from our study illustrate that antibiotics (27.5%) were the most commonly involved medication classes associated with ADRs, followed by antiepileptic medicines (21.6%). This could be due to the wide usage of antibiotics at our study site and based on the number of medications, the chances are high for developing an ADR. Studies report that antibiotics led to 19.0% of emergency department admissions for suspected ADRs in the US between 2004 and 2006 [32]. Another recent study reported that antibiotics (20.8%) are the second most common medication classes associated with ADRs [33].

Wills and Brown classification of ADR reveals that type A (augmented) reactions (76.5%) were most commonly reported, followed by type H (hypersensitivity) reactions (19.6%), which is consistent with literature [34]. Naranjo’s causality algorithm found that most of the reactions had probable relation to the suspected medications (51.0%) followed by possible relation (49.0%), though various measures mentioned in Naranjo’s algorithm were not practically possible at the study site, such as placebo response and drug concentration estimation in body, and these findings would have made a difference in the assessment of causality. Overall, the severity assessment of ADRs using Modified Hartwig and Siegel scale found that most of the ADRs belonged to the moderate (68.6%), followed by mild (21.6%) and severe (9.8%) category. Similar findings were reported by Emma and colleagues from the United Kingdom among 3695 hospitalized inpatients [6].

An Australian study reported that more than 50% of ADR-related hospital admissions were preventable [35]. Similarly, in our study, preventability assessment using Modified Schumock and Thornton scale shows 37.3% of ADRs were probably preventable, while 33.3% were not-preventable and 29.4% were definitely preventable. In addition, our study found that most of the ADRs were predictable (54.9%) and would have been monitored closely to prevent an adverse effect.

Remarkably, our study captured a wide array of ADRs associated with inpatient medication use, and this generated signal would facilitate the healthcare professionals to be vigilant and cautious in prescribing and administering medications to inpatients. The medications that are most frequently associated with ADRs were antibiotics, antiepileptics, antihypertensives and pain medications (Table 3).

In India, epidemiological data concerning ADRs are limited to the incidence, risk factors, outcome and other clinical characteristics. Most Indian studies are based on a single center, small sample and limited duration; hence, they lack representativeness, and it is difficult to extrapolate data from these studies to nationwide. This was one of the limitations of our study. Our study found that by conducting varied ADR assessment scales, we can create awareness about the importance of ADR reporting and thereby promote safer medication use. Similar reporting programs are necessary to educate and to increase awareness about reporting ADRs among the healthcare professionals in some countries.

With the drive to prevent ADRs, interventions that are successful in other countries should be applied in Indian hospital settings, such as implementation of clinical decision support system, computerized medication entry and digital medical record system [36], engagement of full time clinical pharmacists as part of the medical team [37,38] and enabling medication reconciliation during the hospital admission [39]. Numerous ADR risk prediction tools are available and have been published; however, none are globally accepted and are cast off routinely in clinical practice. In future, a sound, widely accepted and validated ADR risk prediction tool is warranted to minimize the risk of ADRs. Newer studies and instruments are warranted to detect preventable ADRs similar to the ‘P Method’ (preventability assessment method) [40]. To further evaluate medication-event causality, MOdified NARanjo Causality Scale for individual case safety reports (MONARCSi) was developed to support pharmacovigilance [41].

## 5. Conclusions

The pattern of ADRs reported by the clinical pharmacy department was comparable with the results from studies conducted elsewhere in a hospital setting. The study was able to showcase the role of clinical pharmacist in monitoring the ongoing safety of medicines through continuous ADR reporting. The findings will encourage the healthcare team to be aware of more ADR-prone medications and their preventability by enhancing the aptitude of prescribers to manage ADRs more effectively. Over half of the reported ADRs are definitely or probably avoidable, and further actions should be taken to present strategies to reduce their impact. Standardized ADR risk prediction tools are useful adjuncts, together with sound clinical judgement underpinned by a trained clinical pharmacist, to monitor ADRs in a hospital setting.

## Figures and Tables

**Table 1:** Characteristics of the study population (*n* = 3157).

Demographic Characteristics		Number of Patients with ADR	Percentage (%)
Sex *	Male	21	42.9
	Female	28	57.1
Age (years) *	≤18	5	10.2
	19–64	23	46.9
	≥ 65	21	42.9
ADR per patient	At least one	48	98.0
	Greater than one	1	2.0

* Chi square test shows not significant at *p* < 0.05.

**Table 2:** Summary of adverse drug reactions based on severity, preventability, predictability, management and outcome.

Parameter	Frequency (*n* = 51)
Classification (Wills and Brown Classification)	
Type A-Augmented	39 (76.5%)
Type B-Bugs	0
Type C-Chemical	1 (2.0%)
Type D-Delivery	1 (2.0%)
Type E-Exit	0
Type F-Familial	0
Type G-Genotoxicity	0
Type H-Hypersensitivity	10 (19.6%)
Type U-Unclassified	0
Causality (Naranjo’s algorithm scale)	
Probable	26 (51.0%)
Possible	25 (49.0%)
Definite	0
Unlikely	0
Severity (Modified Hartwig and Siegel scale)	
Mild-Level 1	0
Mild-Level 2	11 (21.6%)
Moderate-Level 3	25 (49.0%)
Moderate-Level 4 (a)	8 (15.7%)
Moderate-Level 4 (b)	2 (3.9%)
Severe-Level 5	5 (9.8%)
Severe-Level 6	0
Severe-Level 7	0
Preventability (Modified Schumock and Thornton scale)	
Definitely preventable	15 (29.4%)
Probably preventable	19 (37.3%)
Not preventable	17 (33.3%)
Predictability (based on the incidence rate of the reported ADRs as per product information and relevant literatures)	
Predictable	28 (54.9%)
Unpredictable	23 (45.1%)
Management	
Stopped the medication	15 (29.4%)
Substituted another drug	15 (29.4%)
Reduced the dose	12 (23.5%)
Added another drug	9 (17.7%)
Outcome of management	
Recovered	30 (58.8%)
Recovering	21 (41.2%)
Fatal	0

**Table 3:** Drug classes commonly associated with adverse drug reactions.

Drug Class (*n*, %)	Medication	Reaction Details
Antibiotic (14, 27.5)	Ciprofloxacin	Redness and itching
	Metronidazole	Vomiting, Thrombophlebitis
	Ofloxacin	Seizure
	Amoxicillin + Clavulanic acid	Fever
	Cefoperazone + Sulbactam	Inflammation of vein, Diarrhea
	Rifampicin + Isoniazid + Pyrazinamide + Ethambutol	Nausea
	Amoxicillin	Colitis
	Cefotaxime	Itching and rash
	Ceftazidime + Tobramycin	Itching and rash
	Ceftriaxone	Thrombocytosis
Antiepileptic (11, 21.6)	Sodium valproate	Alopecia
	Carbamazepine	Fatigue, Blurred vision, Ataxia
	Clobazam	Memory loss, Hypersomnia
	Topiramate	Anorexia
	Levetiracetam	Decreased appetite
	Oxcarbamazepine	Hypersomnia and fatigue
Antihypertensive (4, 7.8)	Bisoprolol, Carvedilol	Increased breathlessness and QT prolongation
	Furosemide	Hyponatremia, Hypokalemia
	Telmisartan, Cilnidipine	Hypotension
NSAID (3, 5.9)	Ketorolac	Periorbital edema, Redness and itching
	Mefenamic acid	Gastritis
Antipsychotics (2, 3.9)	Olanzapine + Quetiapine	Extra pyramidal symptoms
	Quetiapine	Fatigue
Statin (2, 3.9)	Atorvastatin	Myopathy
	Rosuvastatin	Myopathy
Antifungal (2, 3.9)	Amphotericin B	Renal failure, Diarrhea
Anesthetic (3, 5.9)	Bupivacaine	Post spinal headache *
Cognition enhancer (1, 2.0)	Cerebroprotein hydrolysate	Itching
Antidiabetic (1, 2.0)	Glimepiride	Hypoglycemia
Anti-craving agent (1, 2.0)	Acamprosate	Diarrhea
Antiplatelet (1, 2.0)	Clopidogrel	Abdominal pain
Diagnostic agent (1, 2.0)	Iopromide	Anaphylactic shock
Antianginal (1, 2.0)	Isosorbide dinitrate	Hypotension
Antineoplastic (1, 2.0)	Rituximab	Shivering and breathing difficulty
Others (3, 5.9)	Prazosin	Generalized maculopapular rashes
	Paracetamol	Nephropathy
	Insulin, Furosemide	Hypokalemia

NSAID = Nonsteroidal anti-inflammatory drugs; * = more than once.

## Supplementary Materials

The following are available online at http://www.mdpi.com/2226-4787/6/4/108/s1, Table S1: Thank You note, Table S2: Adverse Drug Reaction documentation form, Table S3: Adverse Drug Reaction Notification Form.
