# Supplementary material for: Study on the Classification, Causality, Preventability and Severity of Adverse Drug Reaction Using Spontaneous Reporting System in Hospitalized Patients
Source: Pharmacy (Basel). 2018 Sep 29;6(4):108. doi: 10.3390/pharmacy6040108 (PMC6306913; doi:10.3390/pharmacy6040108)
Supplement: Supplementary file 1 [file pharmacy-06-00108-s001.pdf]

Supplementary Table 1. Thank You note.

To,

____

____

Dear Dr…,

With your help and support we were able to assess, evaluate and document about the Adverse Drug Reaction (ADR) identified in a patient in your ward. We are hereby briefing the details of the ADR report below.

1. **Patient details:**

   Name: Age: Gender: Ward:

   I.P/O.P No: Date of Admission/Consultation:

   Diagnosis:

2. **Reported Adverse Drug Reaction:** _____________________________________
3. **Suspected drug/s:** ____
4. **Onset of time and date of ADR:** _______________________________________
5. **Classification of ADR:** ____
6. **Predisposing factor:** ____
7. **Causality assessment:** ____
8. **Severity assessment:** ____
9. **Preventability:** ____
10. **Management:** ____
11. **Outcome:** ____
12. **Date and Time of Recovery:** ____

We officially thank you for your esteemed participation in the ADR Reporting. With appreciation for your contribution we request your good self for your constant support and advice which would be valuable for the betterment of the patient care.

Clinical Pharmacist In- Charge:

Supplementary Table 2. Adverse Drug Reaction documentation form.

Patient Details **Report Form No:**

Patient name: Age: Gender:

Weight: Ward: I.P. /O.P. No:

Date of Admission/Consultation:

Consultant/ Physician in- Charge:

Patient's Address:

Reason for admission:

Diagnosis:

Drugs Prescribed:

Sl. No.	Generic Name, Dosage and Strength	Route of Adm	Frequency	Date started	Date stopped	Indication
1<br>2<br>3						
**Suspected Drug/s** (Specify Brand Name, Manufacturer Batch No. & Expiry date)						
1<br>2						

Date of ADR Reporting:

Brief Description of Adverse Drug Reaction:

Onset of reaction: Duration of reaction:

**Past Medical History and Known Allergies**	**Laboratory Data**

Predisposing Factors:

☐ Age ☐ Gender ☐ Genetic ☐ Intercurrent Disease ☐ Multiple Drug Therapy

Additional Note:…………………………………………………………………………………………

………………………………………………………………………………………………...

Seriousness of Adverse Drug Reaction:

☐ Minor side – effect ☐ Required intervention to prevent further complication

☐ Prolonged hospitalization ☐ Disability ☐ Congenital anomaly

☐ Life threatening ☐ Patient Death

☐ Others (Specify): ………………………………………………………………….…..

Management of Adverse Drug Reaction:

☐ Suspected Drug Discontinued ☐ Dose Altered

☐ Treatment/Antidote Prescribed ☐ No Change

Brief on the Management:

Outcome of Management:

☐ Recovered Time/Days taken for recovery:

☐ Recovering

☐ Continuing the same condition

☐ Fatal Time of death:

Any additional note: ………..……………………………………………………...…………..

Challenge strategy:

**Dechallenge** ☐ Yes ☐ No ☐ Do Not Know<br>If Yes, ☐ Definite improvement<br>☐ No Improvement<br>☐ Unknown	**Rechallenge** ☐ Yes ☐ No ☐ Do Not Know<br>If Yes, ☐ Recurrence of symptoms<br>☐ No recurrence of symptoms<br>☐ Unknown

**Reporter Details**	**Consultant/ Physician In- Charge**
Name, Profession & Address:	Name & Address:
Contact No:	Contact No:
Signature:	Signature:

Name & Signature of the Clinical Pharmacist in- Charge:

Supplementary Table 3. Adverse Drug Reaction Notification Form.

**Adverse Drug Reaction Notification Form**	**Date:**
**Patient Name:** **Age:** **Sex:**<br>**Weight:** **I.P. /O.P. No:** **Ward:**	
**Suspected drug/s:**<br>__________<br>__________<br>**ADR observed:**<br>__________<br>**Time and day of ADR:**<br>**Reported by**: ☐ Physician ☐ ☐ Nurse Pharmacist<br>☐ Others(Specify):	
**Name and Signature of the Reporter with Date:**	
Please return this form to the Clinical Pharmacist,<br>**Drug Information Center (address)**<br>**E. Mail. I.D:** **Office:**<br>**Mob.**	
